# LncRNAs Stand as Potent Biomarkers and Therapeutic Targets for Stroke

**DOI:** 10.3389/fnagi.2020.594571

**Published:** 2020-10-19

**Authors:** Junfen Fan, Madeline Saft, Nadia Sadanandan, Bella Gonzales-Portillo, You Jeong Park, Paul R. Sanberg, Cesario V. Borlongan, Yumin Luo

**Affiliations:** ^1^Institute of Cerebrovascular Disease Research and Department of Neurology, Xuanwu Hospital of Capital Medical University, Beijing, China; ^2^Beijing Geriatric Medical Research Center and Beijing Key Laboratory of Translational Medicine for Cerebrovascular Diseases, Beijing, China; ^3^Department of Neurosurgery and Brain Repair, University of South Florida Morsani College of Medicine, Tampa, FL, United States; ^4^Beijing Institute for Brain Disorders, Capital Medical University, Beijing, China

**Keywords:** ischemic stroke, hemorrhagic stroke, long non-coding RNAs, biomarkers, therapeutics

## Abstract

Stroke is a major public health problem worldwide with a high burden of neurological disability and mortality. Long noncoding RNAs (lncRNAs) have attracted much attention in the past decades because of their newly discovered roles in pathophysiological processes in many diseases. The abundance of lncRNAs in the nervous system indicates that they may be part of a complex regulatory network governing physiology and pathology of the brain. In particular, lncRNAs have been shown to play pivotal roles in the pathogenesis of stroke. In this article, we provide a review of the multifaceted functions of lncRNAs in the pathogenesis of ischemic stroke and intracerebral hemorrhage, highlighting their promising use as stroke diagnostic biomarkers and therapeutics. To this end, we discuss the potential of stem cells in aiding lncRNA applications in stroke.

## Introduction

Ischemic stroke is primarily characterized by insufficient blood flow to the brain, whereas hemorrhagic stroke leads to bleeding in the brain, with both types of stroke accompanied by a multitude of biological processes including oxygen deprivation, inflammatory response, oxidative stress, neurotoxicity of excitatory amino acids, apoptosis and edema formation, eventually culminating to severe neurovascular damage (Sahota and Savitz, 2011; Ahad et al., 2020; Virani et al., 2020). Intravascular thrombolysis and mechanical thrombectomy have significantly improved the prognosis of patients with acute ischemic stroke (AIS; Hacke et al., 1995; Berkhemer et al., 2015). However, only a small proportion of patients can receive these two treatments in clinical scenarios due to the narrow therapeutic time window and a considerable incidence of intracranial hemorrhagic conversion (Fonarow et al., 2011; Powers et al., 2018). Little is known about the pathogenesis and treatment options of intracerebral hemorrhage. Surgical evacuation is the main therapeutic option for intracerebral hemorrhage (Gross et al., 2019). Although great efforts have been made in investigating the complex mechanism of stroke-induced neuronal death, there is still a lack of effective treatment for neurological deficits caused by stroke. Therefore, the discovery of new targets and biomarkers is of great significance for ischemic stroke and intracerebral hemorrhage therapy.

**GRAPHICAL ABSTRACT:**
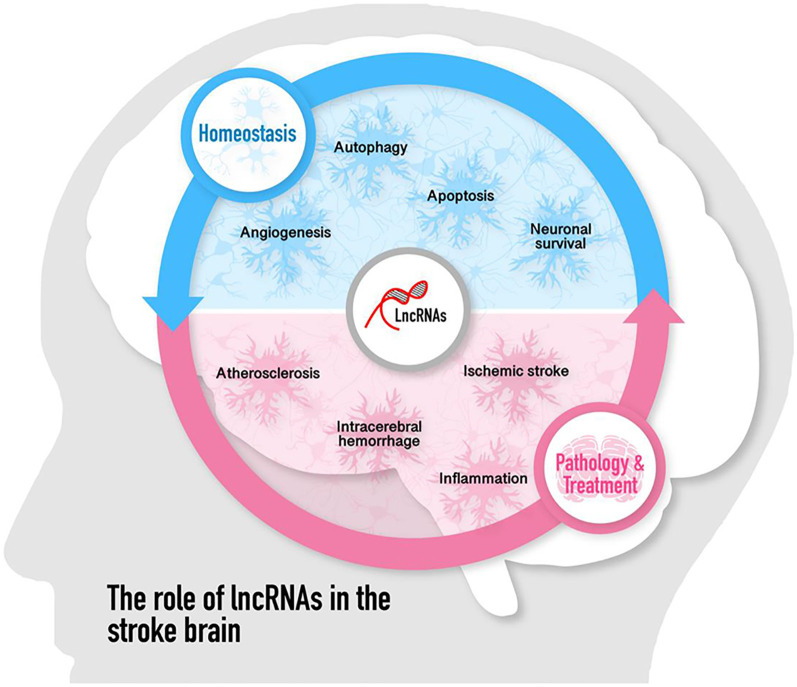


According to the Human Genome Project study, only a minor portion of the mammalian genome encodes the protein-coding transcriptome, whereas the vast majority (approximately 80%) is transcribed into non-coding RNAs (ncRNAs; Djebali et al., 2012). Long noncoding RNAs (lncRNAs) are transcripts longer than 200 nucleotides without or with the ability to encode proteins, but occupy a large part of the transcriptional output (Anderson et al., 2015; Matsumoto et al., 2017; Kopp and Mendell, 2018). LncRNAs were primarily considered as the “noise” of genomic transcription and had no biological functions (Ponting et al., 2009). Mounting evidence illustrates that lncRNAs are essential regulators of various biological processes including cell differentiation, proliferation, and apoptosis, as well as diseases such as cancer, metabolic disorders, cardiovascular diseases, and neurological disorders (Kurian et al., 2015; Bhan et al., 2017; Andersen and Lim, 2018; Yang Q. et al., 2018). LncRNAs also participate in brain development, neuron function, and progression of neurodegenerative diseases (Wan et al., 2017). Recently, lncRNAs have been highlighted to be involved in the pathological process of stroke.

A microarray profiling study firstly showed that lncRNA expression changes in the cerebral cortex at multiple time-points of reperfusion following transient middle cerebral artery occlusion (MCAO) in the adult rat (Dharap et al., 2012). Thereafter, altered lncRNA expression profiles have been investigated in rodent focal cerebral ischemia models, ischemic stroke patients as well as *in vitro* cultured cells following oxygen and glucose deprivation/reoxygenation (OGD/R; Zhao et al., 2015; Zhang et al., 2016; Guo et al., 2018). Kim et al. (2019) investigated the expression pattern of lncRNA from two different intracerebral hemorrhage rat models. The aberrant expression of lncRNAs uncovered in stroke has led researchers to explore the potential roles and mechanisms of specific lncRNA in brain physiology and pathology. We and other groups are among the first to elaborate on the functional significance of individual lncRNA in ischemic stroke. A recent study showed that the expression of lncRNA MEG3 was significantly upregulated following ischemia *in vitro* and *in vivo*, and it mediates ischemic neuronal death by targeting the miR-21/PDCD4 signaling pathway (Yan et al., 2017). LncRNA MALAT1 exerted neuroprotective effects in the post-stroke cerebral microvasculature, resulting in reduced cerebral vascular and parenchymal damage (Zhang X. et al., 2017). Our group reported that compared with healthy controls, circulating lncRNA H19 levels were upregulated in patients with stroke. Knockdown of lncRNA H19 could decrease infarct volume, brain edema, and neuroinflammation by driving HDAC1-dependent M1 microglial polarization (Wang et al., 2017a). Moreover, lncRNA H19 induces cerebral ischemia-reperfusion injury *via* activation of autophagy through the DUSP5-ERK1/2 axis (Wang et al., 2017b). Taken together, these findings indicate that lncRNAs play multiple roles in the post-stroke brain and provide evidence for their importance in stroke pathophysiology.

To date, an increasing number of lncRNAs have been identified to be involved in the molecular processes of ischemic stroke and intracerebral hemorrhage cascade. Here, we provide a systematic and comprehensive summary of the existing knowledge of lncRNAs and stroke in hopes to further elucidate this new research area.

## Mechanisms of LncRNAs Functions

Historically, many of the non-protein-coding parts of the human genome have been considered as junk DNA. However, the development of high-throughput technologies including next-generation sequencing over the past decades allows the in-depth study of noncoding genomes with unprecedented resolution and scale. LncRNAs are defined as transcripts of greater than 200 nucleotides. Most of them cannot be translated into proteins while a few of them encode small peptides (Huang et al., 2017; Kopp and Mendell, 2018). The number of lncRNAs identified is much lower than that of coding protein genes (mRNAs), however, they have higher tissue and organ specificity. Based on the relative position on the chromosome, lncRNAs can be classified into sense lncRNAs, antisense lncRNAs, intronic lncRNAs, intergenic lncRNAs, divergent lncRNAs, promoter upstream lncRNAs, promoter-associated lncRNAs, and transcription start site-associated lncRNAs (St Laurent et al., 2015). LncRNAs are localized in the nucleus or the cytoplasm where they may regulate gene expression at transcriptional or posttranscriptional levels, respectively. Cytoplasmic lncRNAs can stabilize ribonucleoprotein complexes, regulate mRNA stability, or act as competing endogenous RNAs (ceRNAs) for the binding of miRNAs. By contrast, lncRNAs localized to the nucleus regulate gene expression in various modes such as sequestering transcription factors/protein complex away from chromatin (decoy), bringing together distinct proteins to form ribonucleoprotein complexes (scaffold), transcription in response to stimulation or signaling pathway (signal), or acting as a guide by recruiting chromatin-modifying enzymes to target genes (guide) and inducing chromosomal looping to increase the association between enhancer and promoter region (enhancer; Bär et al., 2016). Additionally, lncRNAs can interfere with DNA and form RNA-DNA triplex (Kuo et al., 2019). Because of these diverse and pervasive mechanisms, lncRNAs offer a unique perspective to understand gene regulatory networks. There has been a great deal of research on lncRNAs in different diseases such as cancer, cardiovascular diseases, immune system diseases, and the function of lncRNAs in stroke now is emerging (Taft et al., 2010).

Interestingly, a single lncRNA can modulate multiple targets while one target might be regulated by different lncRNAs, resulting in crosstalk signaling pathways in the network regulated by specific lncRNAs. Therefore, clarifying the molecular mechanism of these crosstalk signaling pathways in the progression of ischemic stroke may reveal key cell death and survival signaling pathways in the brain.

## LncRNA Expression in Ischemic Stroke

LncRNAs are highly expressed in mammalian brain tissues (Mercer et al., 2008). LncRNA expression was first profiled in the cerebral cortex of adult rats at multiple time-points of reperfusion following transient MCAO. Three hundred and fifty-nine lncRNAs were identified to be significantly upregulated and 84 were downregulated at 3 to 12 h of reperfusion following MCAO compared to sham (Dharap et al., 2012). In the following study, they identified that stroke-induced lncRNAs combined with the chromatin-modifying protein (CMP) Sin3A and corepressors of the RE-1 silencing transcription factor (coREST) to regulate the epigenetic transcriptome post-ischemia (Dharap et al., 2013). Liu et al. (2018) demonstrated that a total of 255 lncRNAs (217 upregulated and 38 downregulated) and 894 mRNAs (870 upregulated and 24 downregulated) were significantly differentially expressed in the ischemic brain compared with sham controls. Also, they found that the alteration of lncRNAs expression was closely related to changes in mRNA expression involved in neuroinflammation, cell cycle, cell differentiation, and apoptosis (Liu et al., 2018). Another study used unbiased high-throughput RNA-seq to evaluate the genome-wide expression of lncRNAs in the mouse cortex following focal ischemia. A total of 259 lncRNA isoforms at 6 h, 378 isoforms at 12 h, and 217 isoforms at 24 h of reperfusion were differentially expressed vs. sham controls. Of these, 213, 322, and 171 isoforms at 6, 12, and 24 h of reperfusion, respectively, were novel lncRNAs (Bhattarai et al., 2017). More recently, the group of Frank R Sharp investigated the differential lncRNA expression signatures in peripheral blood samples of male and female humans after stroke. They observed that 299 lncRNAs were differentially expressed between stroke and control males, whereas 97 lncRNAs were altered between stroke and control females. Moreover, some of these differentially expressed lncRNAs mapped closely to previously identified putative stroke-risk genes, suggesting the potential value of lncRNAs as blood biomarkers of ischemic stroke (Dykstra-Aiello et al., 2016). Zhang et al. (2016) initially analyzed differential lncRNA expression in primary brain microvascular endothelial cells (BMECs) after OGD and found that the expression levels of 362 lncRNAs changed significantly. Moreover, they also discovered various conserved transcription factor binding sites in the promoter region of these OGD-responsive endothelial lncRNAs (Zhang et al., 2016). Besides, Deng et al. (2018) identified the expression profile of lncRNAs in peripheral blood mononuclear cells (PBMCs) after AIS and confirmed that linc-DHFRL1-4, SNHG15, and linc-FAM98A-3 were dramatically elevated in ischemic stroke patients compared with healthy controls and transient ischemic attack patients.

The aforementioned studies provide ample evidence that the brain responds to stroke-associated stimuli by significantly altering lncRNAs transcriptomic profiles. This early and robust stroke-induced lncRNA aberration suggests potential functional roles and the predictive values of lncRNAs as new biomarkers for ischemic stroke. A better understanding of the molecular mechanisms after cerebral ischemia will provide an opportunity to explore potential strategies for early diagnosis and therapy of stroke.

## Roles of LncRNAs in the Pathogenesis of Ischemic Stroke

**Figure 1:**
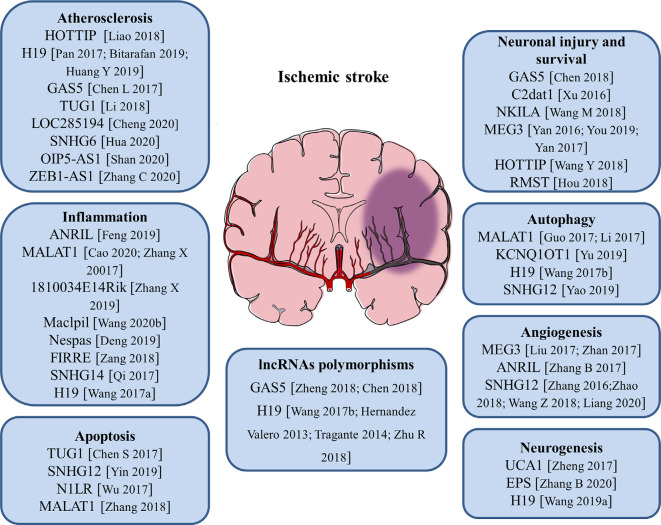
Roles of long noncoding RNAs (lncRNAs) and LncRNA polymorphisms in the pathogenesis of ischemic stroke. The numbers in brackets refer to cited references.

Cumulative studies have indicated that lncRNAs play pivotal roles in regulating the pathological process of ischemic stroke by affecting atherosclerosis, which is a risk factor for stroke, and post-stroke pathophysiology such as inflammation, neuronal injury, and survival (including autophagy and cell apoptosis), angiogenesis as well as neurogenesis. The role of lncRNAs in the pathogenesis of ischemic stroke is discussed in the following paragraphs and summarized in Figure 1.

### LncRNAs in Atherosclerosis

Atherosclerosis is a complex, chronic polygenic disease and the major cause of stroke and heart disease. Endothelial cell proliferation and migration play important roles in the initiation and pathological development of atherosclerosis. Recently, the expression level of lncRNA HOTTIP was found to be upregulated in coronary artery disease (CAD) tissues than in normal arterial tissues. Ectopic expression of HOTTIP promoted endothelial cell proliferation and migration *via* activation of the Wnt/β-catenin pathway (Liao et al., 2018). Located on human chromosome 11, LncRNA H19 is one of the first identified lncRNAs. It has been reported to be upregulated in atherosclerotic patients and regarded as a potential target in the prevention of atherosclerosis and a new biomarker for the diagnosis of atherosclerosis (Pan, 2017; Bitarafan et al., 2019). The study by Huang Y. et al. (2019) revealed that lncRNA H19 contributed to the occurrence of atherosclerosis by promoting ACP5 protein expression and increased the risk of ischemic stroke. LncRNA GAS5 was highly expressed in the plaques of atherosclerosis collected from patients and animal models. Inhibition of lncRNA GAS5 impaired the apoptosis of THP-1 cells after oxidized low-density lipoprotein (oxLDL) stimulation. Moreover, exosomes derived from lncRNA GAS5-overexpressing THP-1 cells enhanced the apoptosis of vascular endothelial cells, suggesting exosomal lncRNA GAS5 participated in the process of atherosclerosis (Chen L. et al., 2017). Compared with healthy controls, lncRNA TUG1 was upregulated in serum samples of patients with atherosclerosis. Gain- and loss-of-function approaches identified that lncRNA TUG1 was closely related to the progression of atherosclerosis (Li et al., 2018). Besides, a variety of lncRNAs including lncRNA LOC285194, lncRNA SNHG6, lncRNA OIP5-AS1, and lncRNA ZEB1-AS1 were proven to be important regulators of atherosclerosis (Cheng et al., 2020; Hua et al., 2020; Shan et al., 2020; Zhang C. et al., 2020). These findings will help to better understand the pathological process of atherosclerosis and aid in the development of novel therapeutic strategies for stroke.

### LncRNAs and Neuroinflammation

At the beginning of an ischemic stroke, the decreased blood flow activates intravascular leukocytes and initiates proinflammatory mediators release, resulting in post-ischemic inflammation. Increasing evidence suggests that inflammatory response is a double-edged sword after stroke, as it not only aggravates secondary brain injury in the acute phase but also has protective effects on brain recovery (Ceulemans et al., 2010). Thus, neuroinflammation is closely related to the progression and prognosis of stroke. Microglia are the main resident immune cells in the central nervous system (CNS) and activated rapidly in response to cerebral ischemia. Stroke-induced brain injury activates microglia and polarizes microglia into pro-inflammatory M1 phenotype or anti-inflammatory M2 phenotype (Hu et al., 2012). Numerous studies showed that lncRNAs are related to neuroinflammation and the activation of microglia in ischemic stroke. It was reported that the expression of lncRNA ANRIL was negatively associated with hs-CRP, TNF-α, and IL-6 levels, but positively correlated with IL-10 levels in AIS patients, suggesting that lncRNA ANRIL play an anti-inflammatory role in AIS progression (Feng et al., 2019). A recent study found that knockdown of lncRNA MALAT1 attenuated the inflammatory injury after brain ischemia, whereas overexpression of MALAT1 exacerbated ischemic brain inflammation (Cao et al., 2020). Besides, silencing or knock-out of lncRNA MALAT1 significantly elevated the expression of proapoptotic factor Bim and proinflammatory cytokines MCP-1, IL-6, and E-selectin in cultured mouse BMECs after OGD as well as in isolated cerebral microvessels in mice after MCAO (Zhang X. et al., 2017). The study by Zhang X. et al. (2019) revealed that the upregulation of lncRNA 1810034E14Rik reduced the expression of inflammatory cytokines not only in ischemic stroke mice but also in OGD-induced microglial cells. Moreover, 1810034E14Rik overexpression could suppress the activation of microglial cells and inhibit the phosphorylation of p65, making it a potential target for stroke treatment (Zhang X. et al., 2019). In monocyte-derived macrophages (MoDMs), lncRNA Maclpil knockdown decreased pro-inflammatory gene expression while promoting anti-inflammatory gene expression, and *in vivo* studies demonstrated that adoptive transfer of macrophages with silenced lncRNA Maclpil robustly reduced infarction and improved behavioral performance of ischemic stroke mice (Wang et al., 2020b). LncRNA Nespas has been reported to play anti-inflammatory and anti-apoptotic roles in cultured microglial cells after OGD stimulation and mice after ischemic stroke by inhibiting TRIM8-related K63-linked polyubiquitination of TAK1 (Deng et al., 2019). Furthermore, lncRNA FIRRE was identified to be upregulated in microglial cells treated with OGD/R and functional experiments revealed that FIRRE upregulation contributed to the OGD/R-induced microglial cell injury through regulating NF-kB (p50)/NLRP3 inflammasome (Zang et al., 2018). The study by Qi et al. (2017) provided the first evidence that lncRNA SNHG14 was highly expressed in ischemic cerebral tissues and OGD-treated microglia. Also, lncRNA SNHG14 increased the expression of PLA2G4A by inhibition of miR-145-5p, which resulted in the activation of microglia in cerebral infarction (Qi et al., 2017). Our group has demonstrated for the first time that circulating lncRNA H19 levels were significantly higher in stroke patients compared with healthy controls. Moreover, this upregulation of plasma lncRNA H19 is positively related to neurological functional deficits and plasma TNF-α levels. Inhibition of lncRNA H19 reduced cerebral ischemic injury and promoted microglial M2 polarization through the downregulation of HDAC1. These findings support an immunomodulatory effect of lncRNA H19 and a novel H19-based diagnosis and therapy for ischemic stroke (Wang et al., 2017a).

Complex mechanisms are involved in the pathophysiology of ischemic stroke, and the inflammatory response plays an important role in its occurrence and development (Tuttolomondo et al., 2012; Anrather and Iadecola, 2016). Once the inflammatory cascade is activated, inflammatory cells secrete various cytotoxic factors such as cytokines, nitric oxide (NO), and matrix metalloproteinases (MMPs) and cause more cellular, blood-brain barrier (BBB), and extracellular matrix damage (Emsley and Tyrrell, 2002; Ouyang, 2013). Therefore, the findings mentioned above may have therapeutic implications and more endeavors are needed to investigate whether pharmacological manipulation of the lncRNAs affects neuroinflammation and stroke outcomes.

### LncRNAs With Neuronal Injuries and Apoptosis

Apoptosis is a programmed, controlled process of cell death, which orderly and efficiently removes damaged cells from DNA damage or during development. Specific cellular signaling pathways and protein synthesis are involved in this self-destruction process (Pistritto et al., 2016). Apoptosis plays an important role in the homeostasis of normal tissues and participates in a variety of clinical diseases. Studies have disclosed that apoptosis may contribute to a significant proportion of nerve cell death following acute brain ischemia, but the underlying mechanisms are still not fully understood. The expression of lncRNA TUG1 increased in the ischemic brain and cultured neurons under OGD insults. LncRNA TUG1 serves as a “sponge” to decrease miR-9 and positively regulates the pro-apoptosis gene Bcl2l11 expression at the post-transcriptional level under ischemia (Chen S. et al., 2017). LncRNA SNHG12 was found to be upregulated in mouse BMECs after cerebral ischemia. The knockdown of SNHG12 inhibited cell proliferation and induced apoptosis under OGD/R condition. Mechanistically, SNHG12 was proven to interact with miR-199a and activated SIRT1 expression, which ultimately led to the activation of the AMPK signaling pathway and attenuated cerebral ischemia/reperfusion injury (Yin et al., 2019). It has been shown that lncRNA-N1LR could enhance cell proliferation, inhibit apoptosis of N2a cells subjected to OGD/R, and reduce neuronal apoptosis and neural cell loss in ischemia/reperfusion (I/R)-induced mouse brains. A mechanistic study showed that lncRNA-N1LR promoted neuroprotection likely through p53 phosphorylation inhibition (Wu et al., 2017). Another study demonstrated that lncRNA MALAT1 regulated MDM2 expression, further restrained the proliferation, and increased the apoptosis of OGD/R induced human BMECs *via* the p53 signaling pathway (Zhang et al., 2018).

### LncRNAs With Neuronal Injury and Survival

Cerebral ischemia/reperfusion post-ischemic stroke induces severe injury to neuronal cells. The role of lncRNAs in neuronal injury or survival has been illustrated. LncRNA GAS5 expression level was upregulated in MCAO-injured brain and OGD-injured primary brain neurons. The knockdown of GAS5 dramatically increased the cell viability and suppressed the activation of caspase-3 and cell apoptosis in neurons subjected to OGD insult. Mechanistically, GAS5 functioned as a ceRNA for miR-137 to decrease neuron survival through inactivating the Notch1 signaling pathway (Chen et al., 2018). Xu et al. (2016) reported a novel cerebral I/R-induced lncRNA, CAMK2D-associated transcript 1 (C2dat1), was augmented in a time-dependent manner in mouse cortical penumbra after focal ischemic brain injury. C2dat1 modulated the expression of CaMKIIδ to promote neuronal survival by activating the NF-κB signaling pathway following cerebral ischemia (Xu et al., 2016). The study by Wang M. et al. (2018) tested the expression and potential function of NKILA (NF-κB interacting lncRNA) in OGDR-treated neuronal cells. They showed that NKILA was upregulated in SH-SY5Y cells and primary murine hippocampal neurons after OGD/R. Silencing of NKILA largely reversed OGD/R-induced neuronal cell viability reduction, apoptosis, and necrosis *via* activating the NF-κB signaling pathway (Wang M. et al., 2018). LncRNA MEG3 was found to be a positive regulator of neuronal death in ischemia and physically and functionally interacted with p53 to mediate ischemic damage (Yan et al., 2016). Repression of lncRNA MEG3 inhibited the neuronal apoptosis, degeneration, and necrosis, enhanced nerve growth, and promoted neurological function restoration after cerebral ischemia-reperfusion injury through the activation of the Wnt/β-catenin signaling pathway (You and You, 2019). Another study reported that lncRNA MEG3 functioned as a ceRNA for miR-21 to regulate PDCD4 expression in ischemic neuronal death following reperfusion. Silencing of MEG3 protected against ischemic damage and improved overall neurological functions *in vivo* (Yan et al., 2017). The expression of lncRNA HOTTIP was decreased in both OGD-injured brain tissues of mice and primary neurons. Gain- and loss-of-function analysis revealed that HOTTIP attenuated OGD-induced neuronal injury and imbalanced glycolytic metabolism. HOTTIP could function as a ceRNA for miR-143 to modulate HK-2 expression and alleviate OGD-induced neuronal injury (Wang Y. et al., 2018). LncRNA RMST silencing is protected against MCAO-induced ischemic brain injury *in vivo* and OGD-induced primary hippocampal neuron injury *in vitro* (Hou and Cheng, 2018). Therefore, lncRNA-mediated epigenetic remodeling of brain/neuronal survival could determine the progression and outcomes of stroke.

### LncRNAs and Autophagy

Autophagy is a highly conserved cellular self-protecting process to degrade misfolded proteins and impaired organelles into metabolic elements and recycle them for cellular homeostasis maintenance. It is widely accepted that autophagy is activated in numerous cell types in the brain such as neurons, glia cells, and microvascular cells upon ischemic stroke (Wang P. et al., 2018). Recent reports suggest that lncRNAs can affect cell survival in stroke by regulating autophagy. LncRNA MALAT1 is one of the most highly upregulated lncRNAs after I/R or OGD/R. It was reported that the knockdown of lncRNA MALAT1 suppressed ischemic injury and autophagy in cerebral cortex neurons after OGD as well as the mouse brain cortex after MCAO. Moreover, MALAT1 served as a molecular sponge for miR-30a and Beclin1 was a direct target of miR-30a (Guo et al., 2017). Another study demonstrated that lncRNA MALAT1 was a potent autophagy inducer, which protected BMECs against OGD/R-induced injury by sponging miR-26b and upregulating ULK2 expression (Li et al., 2017). Furthermore, lncRNA KCNQ1OT1 was highly expressed in the plasma of AIS patients. *In vivo* experiments revealed that inhibition of KCNQ1OT1 remarkably reduced the infarct volume and neurological impairments. Besides, the downregulation of KCNQ1OT1 enhanced cell viability and apoptosis by modulating autophagy in an OGD/R model. Mechanistically, KCNQ1OT1 might target miR-200a to regulate downstream FOXO3 expression, which is a transcriptional regulator of ATG7 (Yu et al., 2019). Additionally, lncRNA H19 induced cerebral ischemia-reperfusion injury *via* the activation of autophagy through the DUSP5-ERK1/2 axis, and OGD/R-induced autophagy activation could be prevented by knockdown of lncRNA H19 (Wang et al., 2017a). The study by Yao et al. (2019) demonstrated that overexpression of lncRNA SNHG12 alleviated OGD/R-induced SH-SY5Y cell injury and induced autophagy activation. On the contrary, inhibition of lncRNA SNHG12 exacerbated neuron cell injury and inhibited autophagy after OGD/R (Yao et al., 2019). These findings allow us to better understand the role of lncRNAs and autophagy in the pathogenesis and development of ischemic stroke.

### LncRNAs and Angiogenesis

Angiogenesis after ischemic brain injury has been discovered to facilitate the restoration of blood supply in the ischemic zone, and higher microvessel density is associated with less morbidity and better functional recovery (Ergul et al., 2012). Post-stroke angiogenesis is a complex biological process that is well-orchestrated by pro-angiogenic and anti-angiogenic factors such as VEGF, BDNF, and bFGF (Liu et al., 2014). Therefore, pro-angiogenesis may represent a potential therapeutic approach that requires more in-depth elaboration for patients with ischemic stroke. Recently, some studies have shown that lncRNAs act as key regulators involved in angiogenesis. Liu et al. (2017) found that lncRNA MEG3 was significantly decreased after ischemic stroke, and silencing of MEG3 increased endothelial cell migration, proliferation, sprouting, and tube formation, resulting in pro-angiogenesis and functional recovery from stroke. Furthermore, they showed that lncRNA MEG3 negatively regulated the notch pathway and inhibition of notch signaling in endothelial cells reversed the pro-angiogenic effect induced by MEG3 downregulation (Liu et al., 2017). Another study also proved that the downregulation of lncRNA MEG3 promoted angiogenesis in OGD/R-induced rat BMECs *via* the p53/NOX4 axis (Zhan et al., 2017). MEG3 may be a potential therapeutic target for promoting regeneration in cerebral infarction and ischemic vascular disease. LncRNA ANRIL could promote VEGF expression and enhance angiogenesis through activation of the NF-κB signaling pathway in rats with diabetes mellitus complicated with cerebral infarction (Zhang B. et al., 2017). LncRNA SNHG12 was significantly increased in OGD-induced primary BMECs and in microvessels isolated from mouse brains after MCAO (Zhang et al., 2016). Another study revealed that lncRNA SNHG12 could promote the angiogenesis in both OGD-injured BMECs and MCAO mouse models *via* regulating miR-150/VEGF signaling (Zhao et al., 2018). Wang Z. et al. (2018) identified that the upregulation of lncRNA SNHG1 promoted angiogenesis by inhibiting miR-199a and regulating HIF-1α and VEGF expression in an OGD/R-induced ischemic stroke model. Moreover, lncRNA SNHG1 functioned as a ceRNA of miR-140-3p to enhance cell proliferation, tube formation, and expression of VEGF, VE-cadherin, and MMP2 through HIF-1α/VEGF signaling (Liang et al., 2020). Undoubtedly, understanding the molecular mechanisms of angiogenesis after ischemic stroke will contribute to the development of pro-angiogenesis therapy for this deadly disease.

### LncRNAs and Neurogenesis

Neural stem cells (NSCs) are a class of self-renewing and multipotent cells. Throughout the process of neural development, NSCs are gradually exhausted and reside in two major neurogenic niches—subventricular zone (SVZ) and subgranular zone (SGZ) in the adult brain. Recent studies revealed that after ischemic stroke, the endogenous quiescent NSCs become active and can differentiate into multiple cell lineages including neuron, astrocyte, and oligodendrocyte, as well as migrate to the ischemic region, which promotes the regeneration of the damaged cells induced by ischemia (Arvidsson et al., 2002; Huang and Zhang, 2019). It was discovered that lncRNAs are involved in the post-ischemic neurogenesis. For example, lncRNA UCA1 enhanced NSCs proliferation, increased nestin expression, and the neurosphere formation through regulating miR-1/Hes1 (Zheng et al., 2017). The study by Zhang B. et al. (2020) developed a novel targeting carrier for microglia with a lncRNA-EPS-loaded liposome. lncRNA-EPS promoted NSCs proliferation and anti-apoptotic ability *in vitro* and lncRNA-EPS-leukosomes improved neuron density in the ischemic core and boundary zone *in vivo* (Zhang B. et al., 2020). One of our studies demonstrated that circulating lncRNA H19 levels were negatively associated with the long-term neurological deficit recovery of patients with ischemic stroke. The knockdown of H19 reduced brain loss and promoted the recovery of neurological deficits through activating p53 to initiate the transcription of Notch1, and finally promoted the process of neurogenesis (Wang et al., 2019a). These studies further strengthen the clinical value of lncRNAs as therapeutic targets for ischemic stroke.

## LncRNAs Polymorphisms and Ischemic Stroke

Clinical epidemiology studies have reported several risk factors for ischemic strokes, such as hypertension, diabetes, obesity, dyslipidemia, and smoking (Yong et al., 2013). Besides, it is widely accepted that genetic factors are also involved in the pathogenesis of ischemic stroke (Dichgans, 2007; Cai et al., 2020). Currently, single nucleotide polymorphisms (SNPs) in some protein-coding genes, including BDNF, apolipoprotein A-V, apolipoprotein B, and ALOX5AP, have been demonstrated to be associated with susceptibility of ischemic stroke (Au et al., 2017; Chen Z. et al., 2017; Bao et al., 2018). Furthermore, lncRNA-related SNPs have been reported to be related to the risk of ischemic stroke (Figure 1). LncRNA GAS5 was observed to be upregulated in ischemic stroke. Zheng et al. (2018) demonstrated that the rs145204276 deletion allele was closely related to the increase of ischemic stroke susceptibility by promoting transcription activity of lncRNA GAS5 and subsequently increasing the expression of GAS5 (Chen et al., 2018; Zheng et al., 2018). LncRNA H19 polymorphisms were reported to participate in the regulation of lncRNA H19 expression. Wang et al. (2017b) found that lncRNA H19 rs217727 and rs4929984 variants were associated with the risk of ischemic stroke and the minor alleles of rs217727 (T) and rs4929984 (A) increased the risk. Moreover, lncRNA H19 rs4929984 was significantly associated with blood pressure. Rs217727 was correlated with coagulation function and homocysteine metabolism of patients with ischemic stroke in the southern Chinese Han population (Huang J. et al., 2019). Recent studies also found that polymorphisms of lncRNA H19 were associated with risk factors including obesity, birth weight, and blood pressure for ischemic stroke patients (Hernández-Valero et al., 2013; Tragante et al., 2014). Additionally, Zhu R. et al. (2018) found that the TT genotype of lncRNA H19 rs217727 polymorphism was statistically associated with an increased risk of ischemic stroke in the northern Chinese Han population. Individuals with the TT genotype had a 1.941 times higher risk of small vessel ischemic stroke when compared with the subjects of CC + CT (Zhu R. et al., 2018; Figure 1).

## LncRNAs in Intracerebral Hemorrhage

**Figure 2:**
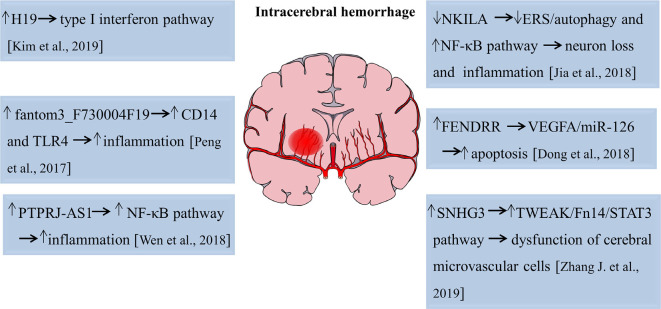
Functions of lncRNAs in the pathophysiological process of intracerebral hemorrhage. The numbers in brackets refer to cited references.

The role of lncRNAs in intracerebral hemorrhage is an attractive field that scientists have tried to elucidate with limited success. Kim et al. (2019) performed microarray analyses and found that 83, 289, and 401 lncRNAs were significantly upregulated, whereas 52, 489, and 786 lncRNAs were significantly decreased of 1, 3, and 7 days after intracerebral hemorrhage, respectively. This study first revealed the temporal expression pattern of lncRNAs with an acute hemorrhagic stroke model induced by stereotactic collagenase injection. Gene ontology/pathway analysis revealed that inflammation is a key biological process after a hemorrhagic stroke and functional study disclosed that lncRNA H19 was the most upregulated lncRNA after intracerebral hemorrhage and closely related to the type I interferon signaling pathway (Kim et al., 2019). Therefore, lncRNA H19 plays an important role both in ischemic stroke and intracerebral hemorrhage. Another study analyzed the expression profiles of lncRNAs and mRNAs in subarachnoid hemorrhage (SAH) brain tissues of mice using high-throughput sequencing. The results showed that approximately 617 lncRNA transcripts and 441 mRNA transcripts were aberrantly expressed at 24 h after SAH. Fantom3_F730004F19, a differentially elevated lncRNA, was identified to be correlated with CD14/TLR4 and increased inflammation in BV-2 microglia cells in SAH (Peng et al., 2017). Also, lncRNA and mRNA microarray revealed a total of 625 lncRNAs, and 826 mRNAs were aberrantly expressed on day 21 after the experimental induction of intracerebral hemorrhage by collagenase. Mitochondrial matrix reduced G-protein coupled receptor activity, and impaired olfactory transduction was discovered to be involved in the late stage of intracerebral hemorrhage (Hanjin et al., 2018). LncRNA PTPRJ-AS1 was highly expressed in inflammatory tissues after intracerebral hemorrhage, activated the NF-κB pathway in microglia, and promoted the secretion of inflammatory cytokines, thereby aggravating inflammatory injury (Wen et al., 2018). Moreover, in type VII collagenase-induced intracerebral hemorrhage rats, silencing of lncRNA NKILA decreased neurological deficits, exacerbated hematoma and brain edema, and induced BBB breakdown, further leading to hippocampal neuron loss and inflammation cytokines production. The endoplasmic reticulum stress (ERS)/autophagy pathway and NF-κB signaling pathway were involved in this process (Jia et al., 2018). Dong et al. (2018) found that lncRNA FENDRR regulated the vascular endothelial growth factor A (VEGFA)/miR-126 axis and contributed to the apoptosis of BMECs in hypertensive intracerebral hemorrhage mice models. LncRNA SNHG3 suppressed the proliferation and migration abilities and increased cell apoptosis and monolayer permeability of BMECs. Pathway analysis revealed that lncRNA SNHG3 impaired cerebral microvascular cells function in intracerebral hemorrhage rats by activating the TWEAK/Fn14/STAT3 pathway (Zhang J. et al., 2019). The functional roles of lncRNAs in pathophysiological changes of intracerebral hemorrhage are summarized in Figure 2. Hence, these studies suggest lncRNAs function as novel targets for the attenuation or exacerbation of brain injuries after intracerebral hemorrhage.

## LncRNAs as Putative Biomarkers and Therapeutic Targets

There are various types of RNA in the circulation regarded as cell-free RNA. Circulating lncRNAs are emerging as promising biomarkers and participate in the screening, diagnosis, progression, treatment, and prognosis of multiple diseases. For example, lncRNA UCA1 in urine has been considered as a noninvasive biomarker of primary bladder cancer (BC; Srivastava et al., 2014). This finding is supported by the fact that UCA1 serves as a possible addition to cystoscopy and cytology treatments when primary BC is detected. UCA1 was identified as a non-coding RNA upregulated in BC compared to normal bladder tissues and is involved in embryogenesis and BC progression (Wang et al., 2006). UCA1 has been studied and confirmed as a biomarker for urothelial carcinoma and expression was increased in various urothelial neoplastic lesions. Increased expression of UCA1 can be associated with tumor proliferation, migration, and invasion. However, the exact mechanisms are still unclear (Srivastava et al., 2014). UCA1 plays an important role in BC pathogenesis and progression and has been shown to promote resistance to cisplatin-based chemotherapy in BC cells (Fan et al., 2014). Furthermore, UCA1 serves as a suitable biomarker in BC and provides significant contributions to other diagnostic procedures.

**Figure 3:**
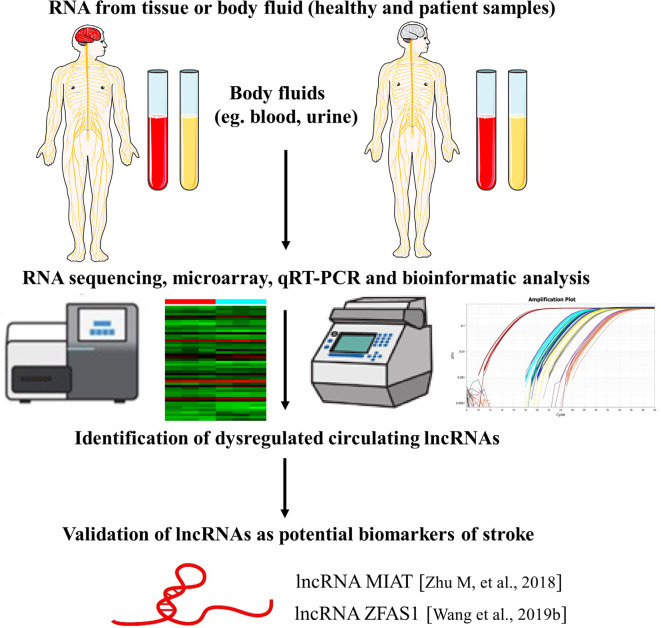
LncRNAs as biomarkers of stroke. The identification of lncRNAs as novel biomarkers of stroke is a multistep process. The figure highlights examples of lncRNA molecules with reported biomarker potential for stroke. The numbers in brackets refer to cited references.

In regards to the use of lncRNAs as novel biomarkers for stroke, a recent study investigated the expression of lncRNA MIAT in the peripheral blood leukocytes of ischemic stroke patients. They found that lncRNA MIAT levels were significantly upregulated in peripheral blood leukocytes of ischemic stroke patients and were associated with stroke severity and infarct volume. Also, higher levels of MIAT were associated with poor prognosis. These findings suggest that lncRNA MIAT in the peripheral blood leukocytes could be used as a novel diagnostic and prognostic marker for ischemic stroke. However, the exact molecular mechanism of MIAT in ischemic stroke and validation with more cases are needed (Zhu M. et al., 2018). Additionally, another study elaborated that lncRNA ZFAS1 levels in the peripheral blood leukocytes were markedly lower in patients with large-artery atherosclerosis (LAA) strokes compared with non-LAA strokes and controls, indicating that lncRNA ZFAS1 has potential predictive value for the diagnosis of LAA stroke (Wang et al., 2019b; Figure 3).

lncRNA may serve as a novel therapeutic strategy for stroke. Elucidating lncRNA’s mechanistic involvement in stroke pathology is key to establishing pharmaceutical treatment. Recent studies have explored specific lncRNAs and their role in stroke-induced neuroinflammation, apoptosis, and oxidative stress. lncRNAs may participate in neuroinflammation *via* macrophage modulation. As previously discussed, Maclpil suppression correlated with diminished expression of pro-inflammatory genes and bolstered expression of anti-inflammatory genes *in vitro*. Also, the delivery of Maclipil *in vivo* spurred a decrease in infarct volume and ameliorated neurological function. Moreover, stifling lncRNA-altered macrophages may bear therapeutic efficacy in alleviating stroke-induced neuroinflammation (Wang et al., 2020b). Also, human primary brain microvascular endothelial cells (HBMVECs) demonstrated truncated lncRNA FAL1 expression following OGD and reoxygenation (Gao et al., 2020). FAL1 overexpression decreased reactive oxygen species (ROS) levels and augmented glutathione (GSH), thereby attenuating oxidative stress. FAL1 overexpression diminished interleukin-6 (IL-6), monocyte chemotactic protein-1, and high mobility group box-1 (HMGB-1) levels, alleviating neuroinflammation. Depleted levels of PAK1 and AKT phosphorylation, along with proliferating cell nuclear (PCNA) numbers, were rehabilitated due to FAL1 overexpression (Gao et al., 2020).

Concerning stroke-induced apoptosis, there seems to be a multitude of various lncRNAs involved. N2a cells exposed to OGD displayed amplified advancement of the cell cycle and N2a proliferation, along with attenuated apoptosis, due to lncRNA-N1LR expression. *In vivo*, lncRNA-N1LR afforded neuroprotection, suppressing apoptosis, and enhancing cell viability, most likely through p53 phosphorylation obstruction (Wu et al., 2017). In parallel, depleting NR-120420 in SH-SY5Y cells exposed to OGD enhanced cell survival and reduced the number of apoptotic cells. *In vivo*, blocking NR_120420 repressed NF-κB phosphorylation, leading to suppressed apoptosis post brain infarction (Wu et al., 2017). Moreover, inhibition of NR_12040 *via* drug therapies may serve as an effective treatment for stroke (Tian et al., 2020). Notably, ischemic stroke samples displayed heightened expression of lncRNA MEG3 and Sema3A and reduced expression of miR-424-5p. When MEG3 was repressed, miR-424-5p was significantly enhanced and Sema3A was downregulated, contributing to bolstered cell survival, hindered apoptosis, and stimulation of the MAPK pathway. In MCAO rats models, the expression level of MEG3 increased gradually at 4, 8, and 24 h point in the process of ischemia-reperfusion, compared to the sham group. MEG3 down-regulated group has smaller ischemic area while it could be reversed by overexpression of Sema3A. Importantly, blocking MEG3 diminished infarct size and ameliorated neurological function, thereby elucidating a therapeutic strategy in stroke (Xiang et al., 2020).

lncRNA H19 has also been shown to make a significant contribution to stroke pathology. In NSCs isolated from the SVZ of rats afflicted with focal cerebral ischemia, H19 lncRNA expression was significantly increased (Fan et al., 2020). H19 knockdown hindered NSC proliferation and differentiation, along with spurring cell death. The elimination of H19 revealed its involvement in the regulation of transcription, apoptosis, cell proliferation, and reaction to ischemia post-stroke. While blocking of H19 enhanced the transcription of genes associated with the cell cycle like p27, H19 overexpression drastically downregulated the transcription of cell cycle-related genes. Also, H19 was shown to modulate miRNAs associated with neurogenesis. Notably, functional rehabilitation following stroke was retarded when H19 was blocked in NSCs (Fan et al., 2020). In another investigation, the sensorimotor cortex displayed upregulation of lncRNA H19 expression after MCAO. Corticospinal axons located in the spinal cord demonstrated bolstered midline-crossing sprouting due to H19 deletion, leading to ameliorated motor function (Hu et al., 2020). Altogether, these studies support the concept that pharmaceutical intervention regulating H19 expression may be therapeutically beneficial in stroke recovery.

In tandem with examining the function of lncRNA in stroke pathogenesis, potential modes of delivery for lncRNA drug therapy warrant further investigation to establish optimal stroke treatment. There is evidence suggesting the efficacy of exosomes in dispatching lncRNA-based therapeutics. Exosomes isolated from human UMSCs impeded cardiovascular malfunction spurred by aging, most likely *via* the secretion of lncRNA MALAT1, suppressing the NF-κB/TNF-α pathway (Zhu et al., 2019). Exosomes extracted from adipose-derived stem cells (hASCs) that carry lncRNA MALAT1 displayed substantial regenerative potential in traumatic brain injury (TBI) by attenuating inflammation. TBI-exposed rats intravenously treated with hASC-derived exosomes revealed that the exosomes were able to penetrate the brain and induced rehabilitation of motor function. Rats treated with exosomes that lacked MALAT1 displayed weaker regenerative capabilities, indicating MALAT1’s pivotal role (Patel et al., 2018). Another study indicated that exosomes derived from adipose stem cells conditioned in hypoxia (HExos) were therapeutically beneficial in ameliorating SCI due to the heightened expression of lncGM37494. LncGM37494 delivery suppressed miR-130b-3p and enhanced expression of PPARγ, leading to greater microglial M1/M2 polarization, which likely contributed to HExos-induced SCI recovery (Shao et al., 2020). These studies support the use of exosomes as conveyers of lncRNA therapeutics, necessitating further investigations of exosome-lncRNA interactions in the context of stroke.

Along the lines of enhancing lncRNA delivery into the brain, liposomes and nanoparticles have also been demonstrated to exhibit as potent carriers of lncRNAs. Long intergenic noncoding RNA bearing (lincRNA-EPS) leukosomes enhanced proliferation of NSCs *in vitro* and hindered apoptosis by repressing activated microglia-induced translocation and cytotoxicity. In transient middle cerebral arterial occlusion (tMCAO) models, penetration of inflammatory cells and cytotoxicity were reduced by lincRNA-EPS leukosomes, and neuronal volume was fortified in ischemic regions (Zhang B. et al., 2020). Regarding nano-based therapeutics, a nano-polymer Meg3 short hairpin RNA plasmid, designed to block Meg3, was conjugated with the OX26 antibody (MPO). *In vitro*, MPO heightened endothelial cell migration and tube generation. *In vivo*, MPO moved to the site of injury and diminished infarct volume, along with the increased expression of Vegfa and Vegfr2 genes, which are related to angiogenesis (Shen et al., 2018). In sum, finding safe and effective carriers of lncRNA that facilitate the proteins’ bioavailability in the brain may potentiate lncRNA’s therapeutic effects in stroke.

Genomics has illuminated particular genetic variants that are correlated with stroke pathology (Ferreira et al., 2019). Variants present in 35 genetic loci have been associated with a higher risk of stroke (Dichgans et al., 2019). However, ischemic stroke does display genetic heterogeneity, as various populations exhibit different genetic variants (Ferreira et al., 2019). Therefore, although evidence has pointed to lncRNA as a promising therapeutic strategy and diagnostic marker for stroke, disparities among individuals must be taken into account due to genotype heterogeneity. For instance, those who are genetically predisposed to have higher levels of circulating cytokines are more susceptible to stroke (Georgakis et al., 2019) and this must be considered when examining stroke outcomes following treatment with lncRNA therapeutics. Also, lncRNAs have numerous genetic variants, whose expression may vary among individuals and affect stroke susceptibility and pathogenesis. SNPs of MALAT1 may influence vulnerability to ischemic stroke. It was found that a variation of the MALAT1 promoter, rs1194338 C > A, could be linked to ischemic stroke susceptibility. Interestingly, those carrying the rs1194338 AC/AA genotypes may have greater resistance to ischemic stroke (Wang et al., 2020a). A study found that the genotype allocation of rs2383207, a variant of lncRNA ANRIL, differed among males with ischemic stroke and control groups (Yang J. et al., 2018). Interestingly, ischemic stroke patients with the GG genotype of rs1333049 demonstrated greater ANRIL expression than those with the CC or CG genotype. Gender also plays a role, as rs2383207 and rs1333049 expressions were linked to higher stroke susceptibility in males but not females (Yang J. et al., 2018). Moreover, these discrepancies among genotypes of individuals may engender inconsistencies in the lncRNA-based diagnosis and therapeutic outcome in stroke and should be further explored in preclinical and clinical investigations.

## Clinical Trials Supporting LncRNAs as Diagnostic Biomarkers and Therapeutics in Stroke

Recent clinical investigations have examined various lncRNAs in the context of stroke. Evaluation of blood serum from acute minor stroke (AMS) patients revealed substantial upregulation of lnc-CRKL-2 and lnc-NTRK3-4 expression and downregulation of RPS6KA2-AS1 and lnc-CALM1-7. Moreover, these lncRNAs may serve as early diagnostic markers in AMS (Xu et al., 2020). Also, a clinical study analyzed the correlation between H19 expression and risk for ischemic stroke in the Chinese Han population. Greater susceptibility to ischemic stroke could be linked to the expression of TT genotype rs217727 in H19. Moreover, H19 rs217727 bears significant potential as a biomarker for the risk of ischemic stroke (Zhu R. et al., 2018). Another clinical study demonstrated that lncRNA ZFAS1 serum levels in ischemic stroke patients were drastically downregulated compared to controls. Large artery atherosclerosis (LAA) stroke patients displayed much lower ZFAS1 expression than in non-LAA stroke patients (Wang et al., 2019b). In ischemic stroke patients, expression of lncRNA and mRNA changed drastically. Examination of blood serum suggests that ENST00000568297, ENST00000568243, and NR_046084 may serve as biomarkers for the diagnosis of ischemic stroke. Notably, analysis of serum revealed that lncRNAs may contribute to stroke pathology by inducing stroke-related mechanisms and mediating miRNA and mRNA expression (Guo et al., 2018). Further understanding of these processes may aid in establishing therapeutics utilizing ENST00000568297, ENST00000568243, and NR_046084 for stroke therapy.

Despite the positive clinical readouts of lncRNAs, equally compelling evidence indicates caution in the use of lncRNA ANRIL in stroke patients. A clinical trial investigated the relationship between antisense noncoding RNA in the INK4 locus (ANRIL) and high-sensitivity C-reactive protein (hs-CRP) and matrix metalloproteinase-9 (MMP-9) in blood serum of ischemic stroke patients. Patients with ischemic stroke exhibited drastic upregulation of ANRIL, hs-CRP, and MMP-9 in serum when compared to the control. A more severe neurological injury could be associated with higher concentrations of ANRIL, hs-CRP, and MMP expression (Zhang K. et al., 2019). On the other hand, another clinical study found that AIS patients demonstrated lower serum levels of lncRNA ANRIL than the control group. Reduced ANRIL expression could be linked to higher hs-CRP concentrations. ANRIL displayed an inverse relationship with inflammatory cytokines TNF-α and IL-6 and a positive association with IL-10 (Feng et al., 2019). These contradicting clinical findings suggest the necessity for careful application of lncRNA ANRIL in stroke. Whereas clinical trials have revealed lncRNA’s capacity as both a diagnostic biomarker and a therapeutic strategy for stroke, additional preclinical studies are warranted to fully elucidate mechanisms and functions of lncRNA in the disease pathology which may aid in the optimal formulation of their clinical applications that are safe and effective in stroke patients.

## Conclusion and Perspectives

Taken together, the discovery of lncRNAs has completely transformed the way, we view the genetic code and the regulation network of gene expression. The emergence of lncRNAs as regulators of stroke provide novel insights and enhance our understanding of the complex regulatory network of pathological cerebral stroke injury. With the development of new technologies such as RNA-seq, deep sequencing, microarray, and stem cell modality, a bulk of aberrantly expressed lncRNAs were identified during stroke onset and disease progression. Great progress has been made to elaborate on the potential roles of individual lncRNA in ischemic stroke and intracerebral hemorrhage. Elucidating the functions and mechanisms of these lncRNAs under normal and pathological conditions may contribute to identifying biomarkers and novel therapeutic targets of stroke.

However, there are still many challenges in this area. To date, only a very small quantity of lncRNAs have been studied in the pathological process of ischemic stroke and even fewer in intracerebral hemorrhage. Large-scale studies are needed to uncover lncRNA functions. Also, numerous lncRNAs have different isoforms due to splicing and the function of different isoforms may vary. Thus, more attention should be paid to the lncRNA isoforms. Moreover, the function of lncRNAs depends on their unique subcellular localization patterns. Many lncRNAs in the nucleus exhibit distinct nuclear localization patterns while others must be exported to the cytoplasm to carry out their regulatory roles. Stem cell technologies, such as the advent of induced pluripotent stem cells from stroke patients, may allow elucidation of lncRNA localization and function during homeostasis and pathological conditions. Linking lncRNA localization and function has the potential to better understand its specialized functions and cellular roles in depth (Chen, 2016). Given the poor-genetic conservation between species, much more work is needed to elucidate the specific role of lncRNAs in stroke. Furthermore, due to the large size of many lncRNAs, crossing through the BBB will be an important issue to be addressed. Exosomes, liposomes, nanoparticles, and lentivirus might be used for carrying lncRNA-based drugs into the cerebral infarction area (Zeng et al., 2016; Quan et al., 2017; Xiao et al., 2020; Zhang B. et al., 2020). The investigation of lncRNAs in stroke is still in its early stage, but its importance is being unraveled in many preclinical studies. Further investigations are still warranted to reveal lncRNA biological functions to accelerate the progress of lncRNA-based therapeutics for stroke.

## Author Contributions

JF, MS, NS, BG-P, YP, PS, CB, and YL conceptualized, drafted, edited, and finalized this manuscript. All authors contributed to the article and approved the submitted version.

## Conflict of Interest

The authors declare that the research was conducted in the absence of any commercial or financial relationships that could be construed as a potential conflict of interest.

## Abbreviations

AIS: acute ischemic stroke
BBB: blood-brain barrier
BMECs: brain microvascular endothelial cells
CAD: coronary artery disease
ceRNAs: competing endogenous RNAs
CNS: central nervous system
CMP: chromatin-modifying protein
coREST: corepressors of the RE-1 silencing transcription
C2dat1: CAMK2D-associated transcript 1
ERS: endoplasmic reticulum stress
I/R: ischemia/reperfusion
LAA: large-artery atherosclerosis
lncRNA: long noncoding RNA
MCAO: middle cerebral artery occlusion
MMPs: matrix metalloproteinases
MoDMs: monocyte-derived macrophages
mRNAs: messenger RNAs
ncRNAs: noncoding RNAs
NO: nitric oxide
OGD/R: oxygen and glucose deprivation/reoxygenation
NKILA: NF-kB interacting lncRNA
oxLDL: oxidized low-density lipoprotein
PBMCs: peripheral blood mononuclear cells
SAH: subarachnoid hemorrhage
SNPs: single nucleotide polymorphisms
VEGF: vascular endothelial growth factor.
